# Predicted *Trans-*Acting siRNAs in the Human Brain

**DOI:** 10.3390/ijms16023377

**Published:** 2015-02-03

**Authors:** Xiaoshuang Liu, Guangxin Zhang, Changqing Zhang, Jin Wang

**Affiliations:** 1 The State Key Laboratory of Pharmaceutical Biotechnology, School of Life Sciences, Nanjing University, Nanjing 210093, China; E-Mails: xsliunju@sina.com (X.L.); zhangguangxin1234@126.com (G.Z.); 2 College of Horticulture, Jinling Institute of Technology, Nanjing 210038, China

**Keywords:** *trans*-acting small interfering RNA, microRNA, human brain, cascade regulation

## Abstract

Endogenous small non-coding RNAs play pivotal roles in regulating gene expression in eukaryotes. Many studies have investigated the function and molecular mechanism of microRNAs in the development and disease of various organisms via mRNA repression of protein-coding genes. Recent findings indicate microRNAs might trigger the generation of *trans-*acting small interfering RNAs (ta-siRNAs). The interaction among different types of small RNA molecules reveals an even more complicated and elaborate pattern of RNA regulation during gene expression than previously thought. We developed a method for mining ta-siRNA sequences and evaluated the performance of our novel method using data from *Arabidopsis thaliana*. Additionally, using small RNA and degradome data for the human brain, we identified 155 small RNAs that satisfied ta-siRNA characteristics. The *DRAXIN* and *ATCAY* genes, which are preferentially expressed in the human brain, were predicted to be the targets of 12 potential ta-siRNAs.

## 1. Introduction

Endogenous non-coding small RNAs (20–25 nt) that regulate gene expression are important to the growth and development of organisms. Two classes of small RNAs that have been widely studied are microRNAs (miRNAs) and small interfering RNAs (siRNAs). In general, miRNAs are about 21 nt in length, are encoded by eukaryotic nuclear DNA, and are involved in transcriptional and post-transcriptional regulation during gene expression. A given miRNA may have different mRNA targets, and the target mRNA sequence may be subject to regulation by numerous miRNAs. In the nucleus, genes corresponding to a miRNA are transcribed by RNA polymerase II to produce an imperfect, self-complementary, hairpin-loop structure known as Pri-miRNA [1]. The Pri-miRNA structure is cut by Drosha to yield pre-miRNA, which is subsequently transported to the cytoplasm by Exportin 5 and then catalyzed by Dicer to generate a double-stranded fragment known as the miRNA/miRNA* duplex. One of the duplex fragments interacts with the RNA-induced silencing complex to target mRNAs. Some miRNAs inhibit the translation of target genes, and some can cleave target genes between bases 10 and 11 from the 5' end of the miRNA, resulting in two fragments.

The biogenesis of siRNAs and the mechanisms by which they engage in post-transcriptional suppression differ from those of miRNAs. Endogenous siRNAs are generated from inverted or direct repeat sequences [2]. Double-stranded RNAs (dsRNAs) are formed by these repeat sequences—A process initiated by the Dicer enzyme—to form double-stranded RNAs that are about 22 nt and have been designated as siRNAs. One of the strands is subsequently incorporated by the RNA-induced silencing complex and then targets homologous mRNAs for cleavage [3]. In plants and nematodes, some cleaved mRNA fragments might also function as templates for dsRNA synthesis, giving rise to transitive RNA-interfering effects [4,5,6,7,8].

Despite the many differences between miRNAs and siRNAs, they are both induced and processed by proteins during their biogenesis. Peragine *et al.* [9] and Vazquez *et al.* [10] identified a third class of small RNA molecules that are now known as *trans*-acting siRNAs (ta-siRNAs). Their biogenesis is initiated by miRNA-directed cleavage of primary transcripts, thus providing a link between miRNA and siRNA regulation [11,12]. These ta-siRNAs suppress the expression of genes that have little overall resemblance to the genes from which they originated. Two enzymes—RDR6 and SGS3—are essential for the production of ta-siRNAs in *Arabidopsis thaliana*. The biogenesis of certain ta-siRNAs requires dual miRNA cleavage sites [13]. It has been shown that in some cases miRNAs suppress gene expression by directing the cleavage of transcripts. The resulting RNA fragments are further processed by SGS3 and RDR6 (RdRP) to produce a dsRNA molecule; this is then cleaved by DICER-like 4 (DCL4) to produce an array of 21-nt small RNAs. Of these small RNAs, some can further regulate gene expression in a miRNA-like manner by cooperating with the Argonaute protein; these small RNAs are referred to as ta-siRNAs [11,14,15].

Four TAS gene families have been identified in *A. thaliana*: *TAS1* (*TAS1a*, *TAS1b*, *TAS1c*), *TAS2*, *TAS3* (*TAS3a*, *TAS3b*, *TAS3c*), and *TAS4* [11,12,16]. Each *TAS* gene produces a number of ta-siRNAs that can regulate genes whose functions range from RNA editing to cell fate [17,18]. Ta-siRNAs have also been found in other plants, such as moss [14], rice [19,20], maize [19], grapevine [21], apple [22] and peach [23].

The discovery of ta-siRNA molecules has provided a link between miRNA and siRNA regulation. Ta-siRNAs are able to enhance the impact of miRNAs through tandem cascade amplification effects [24]. One or two miRNAs might have a wide effect on genes that might be completely unrelated to their original targets. Because ta-siRNAs were found to have highly efficient modes of regulation, they could be adopted by a wider range of organisms. In animals, RdRP activity, which is key to the production of ta-siRNAs, has been detected [25]. In addition, we found that many small RNA clusters exist in the mRNA sequence of animals. We believe that these new siRNAs also exist in animals and take part in gene regulation. We propose a pipeline to predict the potential ta-siRNAs based on the biogenesis of ta-siRNAs. In our current study, we identified a series of *TAS* genes and ta-siRNAs in the human brain through the use of small RNA and degradome datasets.

## 2. Results and Discussion

### 2.1. A. thaliana ta-siRNA Prediction Analysis

To evaluate the performance of our ta-siRNA prediction pipeline, mRNA, small RNA and degradome datasets of *A. thaliana* were used. We identified six ta-siRNAs and seven *TAS* genes in *A. thaliana* (Table 1). Different *TAS* genes can produce similar or identical ta-siRNA sequences, and differing ta-siRNAs can be generated from a single *TAS* gene. Of the seven *TAS* genes, four were confirmed to be ta-siRNA-generating loci; the other three have been predicted to be potential *TAS* genes [24,26]. Two of these three genes are PPR genes, with some PPR genes previously shown to be *TAS* loci [13,24,26,27,28,29], while some are the targets of ta-siRNAs generated from *TAS1* and *TAS2*. Because of the cascade amplification effect of small RNAs, these might also be *TAS* gene locations. These results suggest a very high specificity of our prediction pipeline.

**Table 1:** Prediction results for *A. thaliana* *TAS* genes and ta-siRNAs.

Predicted TAS Genes ^a^	ta-siRNA Sequence	TAS Gene Description ^b^	References
AT1G63400	TGATCAAAAGGTCTATA	pentatricopeptide repeat protein (PPR)	Prediction [24,26]
AT1G62930	TGATCAAAAGGTCTATA	RNA processing factor 3, RPF3	Prediction [24,30]
AT1G63130	TGATCAAAAGGTCTATA	Tetratricopeptide repeat (TPR)-like superfamily protein	TAS2 [9,11,12,13,24,26,27,29,31]
AT1G62910	TGATCAAAAGGTCTATA	pentatricopeptide repeat protein (PPR)	Prediction [24,26]
AT1G63150	TGTGGAAGTTGCTGTTG	pentatricopeptide repeat protein (PPR)	TAS2 [13,24,26,28,29]
	TGATCTTCAACACAATC	pentatricopeptide repeat protein (PPR)	TAS2 [13,24,26,28,29]
AT2G39675	GTTGCTAATACAGTTAC	TAS1C; other RNA	TAS1C [11,12,24,26,32,33]
	TCTAAGTCCAACATAGC	TAS1C; other RNA	TAS1C [11,12,24,26,32,33]
	TTCTAAGTCCAACATAG	TAS1C; other RNA	TAS1C [11,12,24,26,32,33]
AT2G27400	TTCTAAGTCCAACATAG	TAS1A; other RNA	TAS1A [11,19,26,33,34,35,36,37]
	TCTAAGTCCAACATAGC	TAS1A; other RNA	TAS1A [11,19,26,33,34,35,36,37]

^a^ Names of predicted TAS genes are from TAIR; ^b^ From TAIR.

### 2.2. Predicted ta-siRNAs in the Human Brain

**Figure 1:**
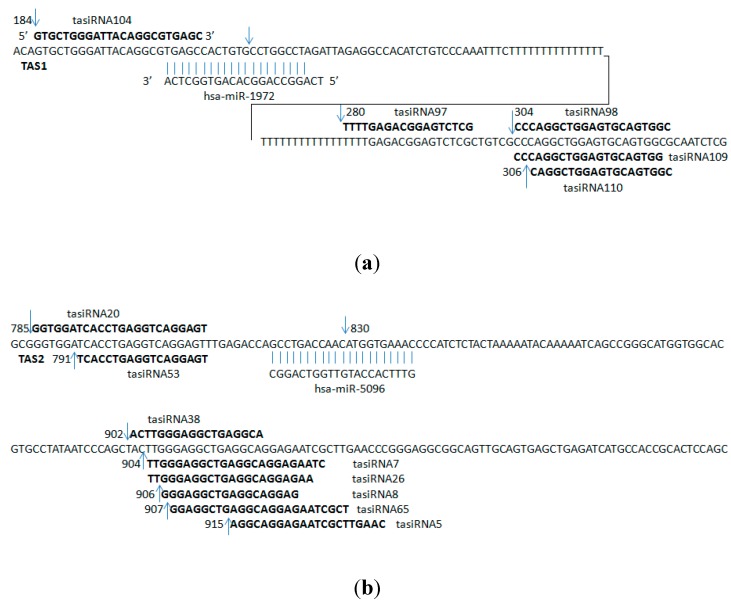
Ta-siRNAs with specific targets site in brain. (**a**) ta-siRNAs targeting DRAXIN, TAS1-hsTAS (ENST00000547850); (**b**) ta-siRNAs targeting ATCAY, TAS2-hsTAS (ENST00000409982). The arrows represent the locations of TAS genes where ta-siRNAs were produced from.

Small RNA and degradome datasets for the human brain were used to predict potential ta-siRNAs. Data were pre-processed by discarding small RNAs with fewer than two reads, and discarding degradome sequences with fewer than ten reads. From our prediction pipeline, 365 small RNA clusters were identified. Of these, 155 potential ta-siRNAs belonging to 324 potential *TAS* genes were predicted. We named the TAS genes after the ensemble transcript ID; for example, if a human TAS gene was at loci ENST00000547850, we named it hsTAS (ENST00000547850), where “hs” indicates *Homo sapiens*. Some of the predicted *TAS* genes were uncharacterized protein genes or non-protein coding genes. The other *TAS* genes were related to an extensive range of metabolic processes, such as vesicle-mediated transport, phosphorylation, phosphate metabolic processes, and ribonucleotide binding. The ta-siRNAs generated from these TAS genes could regulate a wide range of genes and thus have an extensive effect on different aspects of biological processes (Supplementary File 1). The 155 ta-siRNAs are regulated by 13 miRNAs (Supplementary File 2), most of them are poorly conserved in animals. And the predicted targets of these ta-siRNAs were used as search queries in Tissue-specific Gene Expression and Regulation [38,39,40,41]. Two target genes preferentially expressed in the brain were *DRAXIN* and *ATCAY* (Table 2 and Figure 1).

**Table 2:** Uncharacterized *TAS* genes and ta-siRNAs with specific targets in the human brain.

miRNA	*TAS* Gene	ta-siRNA		
	Ensembl Transcript ID	Position	Sequence	No. of Reads
*hsa-miR-1972*	ENST00000547850	184	GTGCTGGGATTACAGGCGTGAGC	3
	ENST00000547850	280	TTTTGAGACGGAGTCTCG	2
	ENST00000547850	304	CCCAGGCTGGAGTGCAGTGGC	2
	ENST00000547850	304	CCCAGGCTGGAGTGCAGTGG	2
	ENST00000547850	306	CAGGCTGGAGTGCAGTGGC	4
*hsa-miR-5096*	ENST00000409982	785	GGTGGATCACCTGAGGTCAGGAGT	2
	ENST00000409982	791	TCACCTGAGGTCAGGAGT	24
	ENST00000409982	902	ACTTGGGAGGCTGAGGCA	2
	ENST00000409982	904	TTGGGAGGCTGAGGCAGGAGAATC	2
	ENST00000409982	904	TTGGGAGGCTGAGGCAGGAGAA	2
	ENST00000409982	906	GGGAGGCTGAGGCAGGAG	3
	ENST00000409982	907	GGAGGCTGAGGCAGGAGAATCGCT	2
	ENST00000409982	915	AGGCAGGAGAATCGCTTGAAC	2

### 2.3. Murine ta-siRNA Prediction Analysis

To confirm our prediction results in the human brain and study the conservation of ta-siRNAs, we compared our findings with those in mice. Degradome data were obtained for the brain, lungs, liver, kidney, ovary and spleen. Small RNA data were from cultured embryonic stem cells [42]. According to distributions of sequence reads for small RNAs and from the degradome data, we removed small RNAs with fewer than two reads. We identified 1038 small RNA clusters. From these clusters, 77 small RNAs from 499 potential *TAS* genes were predicted as potential ta-siRNAs. They are regulated by 24 miRNAs (Supplementary File 2). Similar to the situation in human brain, most of them have a weak conservation. The predicted targets of these ta-siRNAs also had an extensive impact on many biological processes, similar to our findings in the human brain (Supplementary File 3).

### 2.4. Comparison of Human and Murine Predicted ta-siRNAs

The sequencees of three ta-siRNAs in the human brain were similar to a single ta-siRNA in mice (Table 3a). The targets of these ta-siRNAs are related to different aspects of cellular function, such as ion binding, endoplasmic reticulum functions, cell apoptosis, and generation/modification of zinc finger proteins [43,44]. These ta-siRNAs also participate in the FAS and TNFR1 signaling pathways (Table 3b).

Table 3(**a**) Similarities of ta-siRNAs in the human brain and mice; (**b**) Similarity of a mouse ta-siRNA with three human ta-siRNAs.

**Table:** (**a**)

miRNA	*TAS* Gene		ta-siRNA		
	Ensembl Transcript ID	Description	Position	Sequence	No. of Reads
*hsa-miR-5095*	ENST00000338352	OTU domain containing 6A	1382	ATTAGCCGGGCGTGGTGGCA	2
	ENST00000338352	OTU domain containing 6A	1380	AAATTAGCCGGGCGTGGTGGCA	2
	ENST00000338352	OTU domain containing 6A	1382	ATTAGCCGGGCGTGGTGG	3
	ENST00000563601	no protein product	949	ATTAGCCGGGCGTGGTGG	3
	ENST00000574727	no protein product	4185	ATTAGCCGGGCGTGGTGG	3
	ENST00000595787	iduronate 2-sulfatase	5423	ATTAGCCGGGCGTGGTGG	3
	ENST00000340855	iduronate 2-sulfatase	5423	ATTAGCCGGGCGTGGTGG	3
	ENST00000470730	no protein product	2102	ATTAGCCGGGCGTGGTGG	3
*hsa-miR-5585-3p*	ENST00000586372	no protein product	2887	ATTAGCCGGGCGTGGTGGCA	2
	ENST00000586372	no protein product	2885	AAATTAGCCGGGCGTGGTGGCA	2
	ENST00000586372	no protein product	2887	ATTAGCCGGGCGTGGTGG	3
	ENST00000600661	uncharacterized protein	1644	ATTAGCCGGGCGTGGTGG	3
*hsa-miR-1285-5p*	ENST00000599386	p21 protein (Cdc42/Rac)-activated kinase4	5587	ATTAGCCGGGCGTGGTGG	3

**Table:** (**b**)

miRNA	*TAS* Gene		ta-siRNA		
	Ensembl Transcript ID	Description	Position	Sequence	No. of Reads
*mmu-miR-1186a*	ENSMUST00000151163	no protein product	586	AGCCGGGCGTGGTGGCGC	2
	ENSMUST00000030080	sorting nexin family member 30	4190	AGCCGGGCGTGGTGGCGC	2
	ENSMUST00000094892	interleukin 11	1112	AGCCGGGCGTGGTGGCGC	2
*mmu-miR-1186b*	ENSMUST00000146029	no protein product	482	AGCCGGGCGTGGTGGCGC	2
*mmu-miR-3470a*	ENSMUST00000125996	no protein product	3721	AGCCGGGCGTGGTGGCGC	2
	ENSMUST00000124097	no protein product	192	AGCCGGGCGTGGTGGCGC	2
	ENSMUST00000143616	coenzyme Q4 homolog (yeast)	2848	AGCCGGGCGTGGTGGCGC	2
	ENSMUST00000046525	kringle containing transmembrane protein 2	1903	AGCCGGGCGTGGTGGCGC	2
	ENSMUST00000111820	Transmembrane protein with metallophosphoesterase domain	3083	AGCCGGGCGTGGTGGCGC	2
	ENSMUST00000144897	SLX1 structure-specific endonuclease subunit homolog B (*S. cerevisiae*)	1957	AGCCGGGCGTGGTGGCGC	2
*mmu-miR-3470b*	ENSMUST00000179291	no protein product	1868	AGCCGGGCGTGGTGGCGC	2
	ENSMUST00000168013	MAU2 chromatid cohesion factor homolog (*C. elegans*)	4087	AGCCGGGCGTGGTGGCGC	2
	ENSMUST00000050561	MAU2 chromatid cohesion factor homolog (*C. elegans*)	4133	AGCCGGGCGTGGTGGCGC	2
	ENSMUST00000142269	no protein product	4313	AGCCGGGCGTGGTGGCGC	2
*mmu-miR-1196-5p*	ENSMUST00000121443	No protein product	458	AGCCGGGCGTGGTGGCGC	2
*mmu-miR-3473d*	ENSMUST00000098942	SPC24, NDC80 kinetochore complex component, homolog (*S. cerevisiae*)	995	AGCCGGGCGTGGTGGCGC	2

### 2.5. Discussion

The ta-siRNA class of RNA molecules was first identified in *A. thaliana*. Ta-siRNAs produced from *TAS1* and *TAS2* transcripts can target PPRs. There are around 450 PPR-related genes in *A. thaliana*, and these may play a role in RNA editing or binding [45,46]. The PPR genes are also regulated by *miR161.1*, *miR161.2* and *miR1427* in rice [20]. *TAS4* is generated by *miR828* and regulates v-myb avian myeloblastosis viral oncogene homolog (MYB)-related family members, which are also the targets of *miR828*. To date, three *TAS3* loci have been identified in *A. thaliana*: *TAS3a*, *TAS3b*, and *TAS3c* [26]. These three loci regulate auxin response factors (ARFs; also known as tasiARFs), which are signaling molecules that promote the vegetative development of *A. thaliana* from the juvenile to the adult stage. Although only four *TAS* families have been classified, the members of these families have been shown to have remarkable effects on *A. thaliana*. The generation of ta-siRNAs is initiated by miRNAs, and can target genes in a similar way as miRNAs. Thus, the effects of miRNAs can be amplified, resulting in further regulation of target genes. The majority of plant miRNAs function through mRNA cleavage, and several mammalian miRNAs employ this mechanism of action. The siRNA-like mode of plant miRNAs action may therefore be an ancestral mechanism. In support, miRNA has been found to frequently regulate targets via cleavage in members of the ancient phylum Cnidaria. Therefore, it is possible that miRNAs in plants and animals share a common ancestry and that these ancestral miRNAs act via slicing. Slicing generally has fewer targets and a stronger effect on target silencing than do other modes of miRNA action [47]. miRNA-ta-siRNA functions in a manner similar to slicing, so we suspected that the miRNA-ta-siRNA mechanism may exist in mammals.

The predicted ta-siRNAs identified in this study result from a small number of reads. Studies of *Caenorhabditis elegans* and *Drosophila melanogaster* RNAi have revealed the transitive RNAi model, in which dsRNA is amplified to ensure the RNAi response is maintained [7,48,49]. Fire *et al.* also found that small amounts of dsRNA can effectively initiate gene silencing [50]. Therefore, ta-siRNA molecules could function as a form of transitive RNAi, amplifying the miRNA effect in a manner similar to that identified in plants. We suspect that ta-siRNAs and their trigger miRNAs, which exhibited few reads in humans and mice, may also exhibit biological functions via some amplification mechanisms during the RNAi cascade. However, the miRNA-directed ta-siRNA cleavage pathway is different from that of general RNAi directed by exogenous dsRNA. It is highly controlled and the initiation of RNAi is highly selective.

The protein complex RdRP appears to be an important component in the generation of plant ta-siRNAs. In higher eukaryotes, such as the fly, human, and mouse, the role of RdRP may be performed by various functional homologs [51,52,53,54]. Furthermore, ta-siRNAs can be bound by Argonaute and function as miRNAs. To assess Argonaute-bound ta-siRNAs, a dataset from mice was used [55]. Of the 77 predicted ta-siRNAs in mice, 12 are in the Argonaute-bound small RNA dataset (Supplementary File 4). This data further support our claim of the existence of mammal ta-siRNAs.

In plants, some ta-siRNAs are from the 21-nt phase positions on the transcripts cleaved by miRNAs, such as ta-siRNAs arising from the *TAS1* loci in *A. thaliana* [11]. However, some ta-siRNAs are from “non-phased” positions on transcripts, such as those from the *TAS3* loci in *Vitis vinifera* [56]. Our prediction pipeline includes both “phased” and “non-phased” situations to achieve better prediction results.

Although research into ta-siRNAs is not as popular as miRNA research, their importance in regulating the relationship between miRNAs and mRNAs is crucial. There are many ta-siRNA-related mechanisms and functions that remain unclear, and are thus potential avenues of future research. Understanding more about ta-siRNAs would likely help us better understand miRNAs and siRNAs, and their interactions. The prediction pipeline we have designed offers a new method to identify potential ta-siRNAs, which could be applied to future studies.

## 3. Experimental Section

### 3.1. Datasets and Tools

Small RNA datasets sequenced by Illumina, and degradome datasets were downloaded from the Gene Expression Omnibus repository (http://www.ncbi.nlm.nih.gov/gds, Supplementary File 5). The miRNA sequences were downloaded from miRbase (release 19) and cDNA sequences were from Ensembl (release 69). Bowtie [57], RNAhybrid [58] and BLASTn [59] were integrated into our prediction pipeline.

### 3.2. Prediction Flow

The principle of prediction is based on the generating process of ta-siRNAs found in plants. The binding sites of human miRNAs on mRNAs were predicted through an imperfect complementary match. RNAhybrid was then used to find the minimum free energy for the hybridization of miRNAs with the potential target mRNAs. According to previous studies [60], the free energy of a microRNA:mRNA duplex below −25 kcal/mol would be a relatively stringent threshold value. Therefore, sites with less than −25 kcal/mol energy were viewed as potential miRNA targets.

The Illumina small RNAs were mapped to mRNA sequences to determine small RNA clusters. According to the mechanism described above, ta-siRNA features a cluster of small RNAs that are derived from the same *TAS* gene. An mRNA region with at least three small RNA hits and a maximum distance of 100 nt between hits was considered a small RNA cluster [61]. The predicted targets of miRNAs were used to screen small RNA clusters for ta-siRNA candidates.

**Figure 2:**
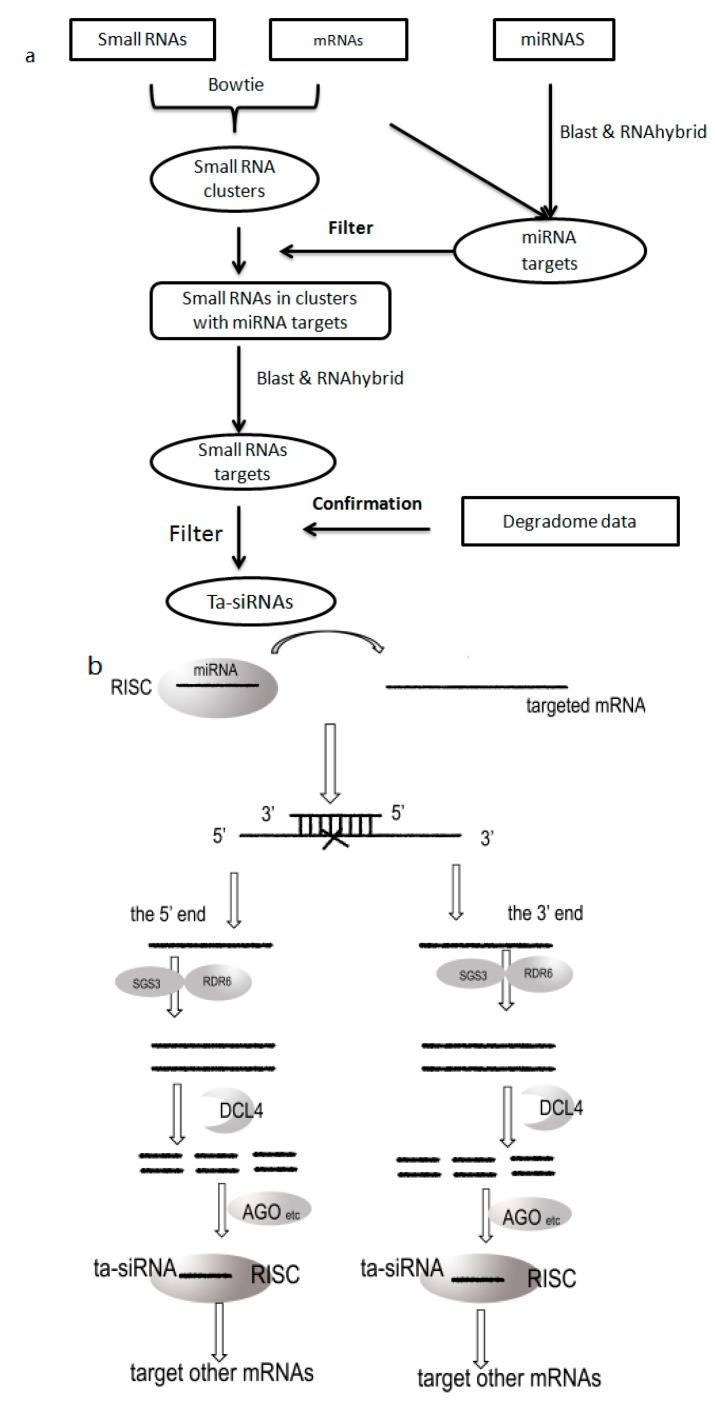
(**a**) Schematic of our ta-siRNA prediction pipeline; (**b**) Generation of ta-siRNAs.

The degradome dataset was used to further ensure the accuracy of our predictions. The targets of small RNAs in the clusters (ta-siRNA candidates) were predicted because ta-siRNAs can amplify tandem cascades with respect to miRNA functions. We used the same parameters that were employed for miRNA target prediction. If fragments cleaved by the ta-siRNA candidate matched the sequence from the degradome data, it was regarded as a potential ta-siRNA. The whole prediction flow is shown in Figure 2.

### 3.3. Model Assessment

We used known *A. thaliana* *TAS* genes to evaluate the performance of our prediction pipeline. The mRNA sequences of the relevant *A. thaliana* *TAS* genes were from The Arabidopsis Information Resource (TAIR; http://www.arabidopsis.org/). We downloaded the small RNA data from Arabidopsis MPSS Plus [62,63], while the degradome data were downloaded from the Gene Expression Omnibus database.

### 3.4. Conservation of ta-siRNAs between Human and Mouse

To investigate the conservation of ta-siRNAs between mice and humans, we predicted murine ta-siRNAs using our pipeline and compared them with known human ta-siRNAs.

## 4. Conclusions

Based on the process by which ta-siRNAs are generated, we designed a prediction pipeline to identify novel ta-siRNAs in humans. As the research continues, many new characteristics of miRNAs have been shown. In our algorithm, we designed a new method to predict the targets of miRNAs. The differences between our method and other target prediction tools are: (1) we do not require the conservation of mRNAs; (2) the targets of miRNAs are not restricted to the 3' UTR; (3) the “seed” sequence can be 1–7 nts. The criteria for target prediction were set based on miRNA properties and experimental experiences. The binding energy of miRNA to its target was below −25 kcal/mol and the least continuous match was 7 bp based on the “seed sequence” of miRNAs. Our prediction pipeline was first applied to *A. thaliana* to estimate its accuracy. Seven ta-siRNAs were predicted, of which four were known ta-siRNAs with the remaining three were predicted by other algorithms. Our prediction results in *A. thaliana* were highly specific. When our prediction analysis was applied to humans, we identified 324 potential *TAS* genes and 155 potential ta-siRNAs. In mice, 499 potential *TAS* genes and 77 potential ta-siRNAs were identified. Among these predicted ta-siRNAs, three ta-siRNAs in humans were similar to a single ta-siRNA in mice. The trigger miRNAs of ta-siRNAs predicted in human brain and mice are also poorly conserved. These findings suggest that ta-siRNAs are weakly conserved, which is in accordance with a recent finding that non-coding genes exhibit relatively low levels of conservation [64].

## Supplementary Materials

Supplementary files can be found at http://www.mdpi.com/1422-0067/16/02/3377/s1.
